# Gut Microbiome of Aquatic Organisms: Role in Immunity Formation and Effects on Aquaculture Productivity

**DOI:** 10.3390/microorganisms14081734

**Published:** 2026-08-07

**Authors:** Liudmila E. Khmelevtsova, Evgeniya V. Prazdnova, Maria S. Mazanko, Yaroslav A. Brislavsky, Dmitry V. Rudoy

**Affiliations:** Research Laboratory «Agrobiotechnology Center», Don State Technical University, 344003 Rostov-on-Don, Russia; prazdnova@sfedu.ru (E.V.P.); mary.bio@list.ru (M.S.M.); brislavskii@yandex.ru (Y.A.B.); dmitriyrudoi@gmail.com (D.V.R.)

**Keywords:** aquaculture, hydrobionts, gut microbiome, probiotics, prebiotics, synbiotics, immune response, short-chain fatty acids, disease resistance

## Abstract

Aquaculture is central to global food security, but intensification of production has increased disease risk and environmental pressure. The gut microbiome of aquatic organisms is now recognized as a key mediator of host physiology, nutrition, immunity, and pathogen resistance, making it a promising alternative to antimicrobial-based disease control. This review summarizes current knowledge on the composition, assembly, and functional roles of the gut microbiome in fish and crustaceans of aquacultural importance. Major bacterial phyla include Proteobacteria, Firmicutes, Bacteroidetes, Fusobacteria, Actinobacteria, and Verrucomicrobia. Community structure is shaped by environment, diet, host age, genetics, stress, and stochastic processes, with differences between marine and freshwater systems. The microbiome contributes to immune defense through short-chain fatty acid production, Toll-like receptor signaling, cytokine regulation, mucosal immunoglobulin responses, antimicrobial peptide and bacteriocin production, and competitive exclusion of pathogens. It also supports productivity by improving nutrient assimilation, vitamin and enzyme synthesis, and feed conversion. Probiotics, prebiotics, and synbiotics are discussed as strategies for targeted microbiome modulation, although unstable colonization and the lack of standardized protocols remain major challenges. Overall, targeted microbiome manipulation offers a promising route toward sustainable, antimicrobial-reduced aquaculture.

## 1. Introduction

Aquaculture is a vital element of the global food system, playing a significant role in ensuring food security worldwide and meeting the increasing demand for animal protein [1]. However, increasing production comes with risks. For example, higher stocking densities can worsen living conditions and lead to disease outbreaks, which reduces the sector’s profitability [2].

In this context, the gut microbiome of aquatic organisms represents an important link connecting host physiology with the external environment. Contemporary research demonstrates that symbiotic bacteria not only participate in nutrient assimilation but also modulate immune responses through the production of short-chain fatty acids (SCFAs), activation of Toll-like receptors (TLRs), and regulation of cytokines (IL-1β, TNF-α, IL-10) [3]. For example, aquatic organisms with high microbiota alpha diversity exhibit increased resistance to colonization by *Vibrio* pathogens [4,5]. Moreover, dysbiosis induced by factors such as antimicrobials can downregulate the expression of immune-related genes in the intestinal tissues of fish [6].

**Figure 1:**
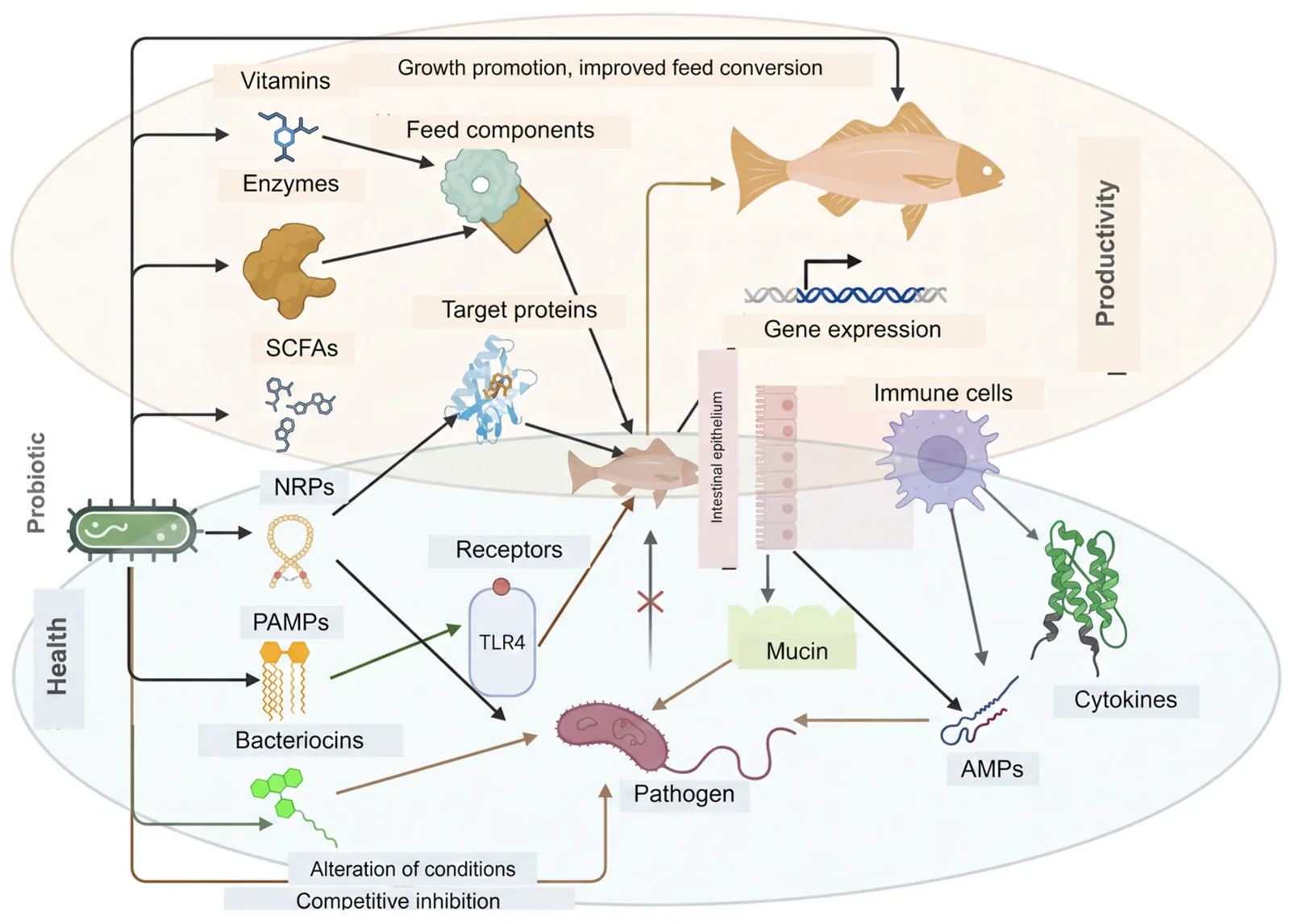
Main mechanisms through which microbiome bacteria affect the health and productivity of aquatic organisms.

Despite progress in understanding the role of microbiota, unresolved questions remain. For instance, the mechanisms of colonization by specific probiotic strains (e.g., *Bacillus*) under open aquaculture system conditions have been insufficiently studied [7]. Furthermore, standardized protocols for the application of synbiotics—combining probiotics and prebiotics—for different fish species and crustaceans are lacking [8]. The main mechanisms through which microbiome bacteria affect the health and productivity of aquatic organisms are presented in Figure 1.

The gut microbiome of aquatic animals has already been examined in several reviews addressing, for example, the microbiome of fish and shellfish in general [9], the microbiota of marine fish specifically [10], or the use of probiotics, prebiotics, and synbiotics in narrower host groups such as decapod crustaceans [11]. The present review is distinguished in three respects. First, it considers fish and crustaceans within a single comparative framework rather than treating them separately, contrasting their microbiome composition, immune interactions, and responses to modulation. Second, it foregrounds the mechanistic link between microbiome-mediated immune formation and aquaculture productivity, rather than describing microbiome composition or probiotic supplementation in isolation. Third, it emphasizes recent (2023–2025) evidence and the translational bottlenecks—unstable colonization in open systems, dose optimization, and multi-omics-guided personalization—that currently limit practical application.

Accordingly, the present review summarizes current knowledge on the composition, assembly, and functional role of the intestinal microbiome of aquatic organisms, evaluates the potential of its targeted modulation to improve the productivity and health of aquaculture species, and outlines promising strategies for implementing these technologies in practice, with particular attention to differences between fish and crustaceans. As a narrative review, the literature was surveyed selectively to illustrate key concepts and mechanisms. The review sequentially addresses the composition and factors governing the formation of the gut microbiome; its role in immune defense against pathogens and in ensuring the productivity of aquatic organisms; practical approaches to microbiome modulation using probiotics, prebiotics, and synbiotics; and current challenges and promising research directions.

## 2. Composition and Formation of the Gut Microbiome of Aquatic Organisms

### 2.1. Microbiome Composition

The gut of aquatic organisms—primarily fish and crustaceans—is colonized by a complex microbial community that plays a key role in digestion, metabolism, immune system development, and resistance to pathogens. The number of bacterial cells in the fish intestine can reach 10^8^ CFU per gram of contents, with diversity estimated at hundreds of species [12]. However, despite this, the gut microbiome of fish is characterized by relatively low phylogenetic diversity compared with other ecosystems. The bacterial community of the fish intestine differs markedly from those of other vertebrate classes (reptiles, birds, and mammals) [13]. The gut microbiome of aquatic organisms is dominated by bacteria belonging to the phyla Proteobacteria, Firmicutes, Bacteroidetes, Fusobacteria, Actinobacteria, and Verrucomicrobia, although their relative proportions can vary substantially depending on host species, age, diet, and environmental conditions [14]. Table 1 presents systematized data on the composition and functional role of microorganisms in the gut microbiome of aquaculture species.

The most prevalent phylum in the intestine of aquatic organisms is Proteobacteria [15]. The second most prevalent phylum, particularly abundant in herbivorous fish species, is Firmicutes; Bacteroidetes is also an important component of the microbiome [9]. Fusobacteria are frequently encountered in the fish intestine, especially during the reproductive period [16]. Actinobacteria and Verrucomicrobia are present in smaller quantities, but are consistent members of the microbiome. Cyanobacteria are also found in the intestines of some fish species [17].

**Table 1:** Major bacterial phyla in the gut microbiome of aquatic organisms (including fish and crustaceans).

Bacterial Phylum	Prevalence	Functional Role	Reference
Proteobacteria	Most prevalent phylum, especially in predatory species	Proteolytic activity, protein degradation	[10,15]
Firmicutes	Second most prevalent, especially in herbivorous species	Carbohydrate fermentation, SCFA synthesis	[9,18]
Bacteroidetes	Important microbiome component, especially in herbivorous fish	Degradation of complex polysaccharides	[9,19]
Fusobacteria	Frequently encountered, especially during the reproductive period	Vitamin and SCFA synthesis	[16,18]
Actinobacteria	Present in smaller quantities	Antimicrobial activity	[10,18]
Verrucomicrobia	Less prevalent but regularly detected	Mucin degradation, maintenance of barrier function	[10,20]
Cyanobacteria	Found in the intestines of some fish species	Photosynthesis, nitrogen fixation	[17,19]

These phyla collectively account for up to 90% of the entire gut microbiome, indicating a certain degree of conservation of the core microbiome composition regardless of host species or environmental conditions [12].

Differences exist in gut microbial community composition between marine and freshwater aquatic organisms. Among the most prevalent genera detected in the intestines of many marine fish species are *Vibrio* and *Photobacterium*; a meta-analysis of gut microbial communities in marine fish demonstrated that bacteria of the order Vibrionales (encompassing the genera *Vibrio* and *Photobacterium*) accounted for up to 70% of all sequences obtained by gut microbiome sequencing [10]. Using juveniles of yellowfin seabream (*Acanthopagrus latus*) as a model, it was demonstrated that when fish were maintained in brackish water (10 ‰), bacteria of the genus *Cetobacterium* predominated in the intestine, whereas marine water (30 ‰) conversely promoted the proliferation of bacteria of the genus *Vibrio* [21].

The genus *Pseudomonas*, which possesses proteolytic activity, is also widely distributed [22]. *Mycoplasma* and *Cetobacterium* have been detected in the intestines of some marine fish species, and *Acinetobacter* and spore-forming *Bacillus* are regularly isolated as well [10]. In shrimp adapted to higher salinity, Proteobacteria were the dominant phylum in the gut microbiome, followed by other bacterial groups (Bacteroidetes, Planctomycetes, and Firmicutes), and the most prevalent genus was *Vibrio*, belonging to the *harveyi* clade [23].

The gut microbiome of freshwater fish differs from that of marine species. Kim et al. [13] found that the phyla Firmicutes and Fusobacteria were significantly enriched in freshwater fish, whereas Proteobacteria was enriched in marine fish. At the family level, Moraxellaceae, Vibrionaceae, and Enterobacteriaceae (all belonging to the class Gammaproteobacteria), as well as Alcaligenaceae (Betaproteobacteria), were significantly more abundant in marine fish than in freshwater fish, whereas Aeromonadaceae (Gammaproteobacteria) were significantly more abundant in freshwater fish. The family Clostridiaceae (Clostridia) was more abundant in freshwater fish than in marine fish, whereas Leuconostocaceae (Bacilli) was more abundant in marine fish. With respect to alpha-diversity indices, the gut microbiota of freshwater fish demonstrated significantly higher values of microbial richness (Shannon index), non-phylogenetic diversity (observed species), and evenness (Simpson’s evenness) than that of marine fish [13]. One of the dominant genera in the intestine of freshwater aquatic organisms is *Aeromonas* [18]. However, the genus *Aeromonas* also includes dangerous pathogens of freshwater fish, primarily *Aeromonas hydrophila*—the causative agent of motile aeromonad septicemia (MAS), which causes ulcerative-hemorrhagic lesions and mass mortality in aquaculture fish—as well as *Aeromonas salmonicida*, which causes furunculosis in salmonids [24,25]. An important bacterial genus in freshwater aquatic organisms is *Cetobacterium*, known for its capacity to produce large quantities of vitamin B12 [19]. It was demonstrated that the microbiota of wild freshwater fish contains a complete set of genes for de novo biosynthesis of vitamin B12, with the genus *Cetobacterium* being the key contributor and possessing probiotic potential [26]. In addition, representatives of the family Enterobacteriaceae have been detected in the intestines of freshwater fish and are also considered an important microbiome component [22].

Microorganisms inhabiting the intestine are of great importance for digestion and other physiological processes of aquatic organisms. The participation of the gut microbiome in nutrient assimilation and immune responses of aquatic organisms will be examined in greater detail below; however, in general terms, the following functionally important groups can be identified. Cellulolytic bacteria, capable of degrading complex polysaccharides from plant-based food, are important for herbivorous fish. The intestinal microbiomes of such fish are typically dominated by Bacteroidetes and Firmicutes, in particular those belonging to the order Clostridia, such as *Epulopiscium* [27]. Studies of the gut microbiota of the herbivorous grass carp (*Ctenopharyngodon idella*) revealed a predominance of cellulolytic bacteria of the genera *Aeromonas*, *Enterobacter*, *Enterococcus*, *Citrobacter*, *Bacillus*, *Raoultella*, *Klebsiella*, *Hydrotalea*, *Pseudomonas*, and *Brevibacillus* [28].

Proteolytic bacteria that produce proteases for protein degradation (e.g., *Cetobacterium* and *Halomonas*) predominate in predatory fish [29]. Some bacteria inhabiting the fish intestine possess probiotic properties and contribute to maintaining host health. Thus, Lv et al. isolated probiotic strains of *Lactobacillus sakei*, *Pediococcus acidilactici*, and *Bacillus subtilis* capable of inhibiting the growth of certain bacterial pathogens from the intestine of the cold-water fish *Scophthalmus maximus* [30]. Bacteria that synthesize vitamins and short-chain fatty acids (*Bacteroides*, *Clostridium*, *Fusobacterium*) play an important role in supplying the host with essential nutrients and regulating its immune response [12].

### 2.2. Conditions and Dynamics of Microbiome Formation

The formation of the gut microbiome of aquatic organisms occurs under the influence of several key factors: environment, diet and trophic specialization, age and developmental stage, species-specific characteristics and host genetics, stress factors, and stochastic processes.

#### 2.2.1. Environment

Microbiome formation begins at early developmental stages; larvae of aquatic organisms acquire their first microorganisms from water and feed [12]. Huang et al. demonstrated in experiments with shrimp that microbial communities of water and bottom sediments in aquaculture systems were the primary sources for gut colonization in aquatic organisms, particularly at early developmental stages [31]. It was found that up to 85% of the gut microbiota of shrimp was formed through migration of microorganisms from water and bottom sediments, with the contribution of each source varying across different rearing stages. However, as the organism grows and the immune system matures, the gut microbiome becomes more host-specific and diverges from the surrounding environment [12]. A comparison of two aquaculture methods—intensive pond culture (IPC) and lotus–fish co-culture (LFC) in the rearing of Hefan crucian carp—demonstrated that aquaculture regimes exerted a substantial influence on the bacterial community of the fish gut. Significantly higher bacterial diversity in the gut was found in LFC compared with IPC [32]. Similar results were obtained for the microbiomes of three cold-water fish species (herbivorous *Schizothorax wangchiachii*, omnivorous *S. kozlovi*, and predatory *Percocypris pingi*) from cultured and wild habitats [33]. The network complexity of the gut microbiota and its alpha and beta diversity were significantly higher in wild cold-water fish than in cultured individuals. In artificially reared fish, Proteobacteria predominated (82%), whereas in wild fish several taxa co-dominated (Proteobacteria (38%), Fusobacteria (30%), Firmicutes (11%), Cyanobacteria (11%)). Significant differences in the gut microbiome composition of wild, domesticated, and aquaculture individuals of *Gymnocypris potanini firmispinatus* were also identified at the phylum and genus levels, reflecting differences between wild-habitat and farm conditions. The highest microbiota richness and alpha diversity were observed in wild individuals, while domesticated and cultured individuals showed lower values [34]. Interestingly, in this case Proteobacteria predominated in the wild group, whereas Fusobacteria and Proteobacteria co-dominated in the domesticated and cultured groups.

In an analysis of the microbiome of 227 fish individuals representing 14 orders, 42 families, 79 genera, and 85 species, Kim et al. [13] found that the gut microbial community of fish was determined more by host habitat than by host taxonomy or trophic level.

#### 2.2.2. Diet and Trophic Specialization

Diet is one of the main factors determining the composition and functional structure of the gut microbiome. In herbivorous fish, bacteria capable of degrading complex polysaccharides predominate (e.g., representatives of Bacteroidetes and Firmicutes, in particular Clostridia), whereas in predatory species, microorganisms specializing in the utilization of proteins and lipids dominate (e.g., Proteobacteria) [27]. Metagenomic studies indicate that even in the presence of a shared “functional core” (fermentation of simple carbohydrates, synthesis of B vitamins, etc.), the microbiomes of fish with different dietary types differ substantially in their enzyme system repertoire: in the bacteria of herbivorous species, the diversity of genes associated with fiber and hemicellulose degradation is higher, while in the bacteria of predatory fish, genes involved in protein and fatty acid degradation are more diverse [35]. The trophic level of the host is weakly but significantly associated with gut microbial community structure. It was found that the variability in the gut microbial community attributable to host taxa was greater at lower taxonomic levels than at higher taxonomic levels [13]. Yang et al. demonstrated that bacterial diversity (Shannon and Simpson indices) in herbivorous fish was significantly higher than in carnivorous fish; *Clostridium* was significantly more abundant in carnivorous fish than in herbivorous fish, while *Niameybacter*, *Alistipes*, *Desulfovibrio*, and *Akkermansia* were more abundant in herbivorous fish [36]. Microbiome composition can serve as an informative indicator of successful dietary adaptation in fish, as was shown for the endangered pallid sturgeon (*Scaphirhynchus albus*) upon release of individuals into the wild [37]. However, in some cases, no significant change in gut microbiome composition has been observed upon diet change. For example, when either fish meal or spirulina was added as a protein source to the diet of African catfish, no substantial differences in the species composition of the gut microbiome were detected, and the most prevalent bacteria in both experimental groups were Fusobacteriia [38].

#### 2.2.3. Age and Developmental Stage

The microbiome becomes more stable and resilient to external influences as the aquatic organism matures. In silver crucian carp (*Carassius auratus gibelio*), the diversity of the gut microbiota increased significantly over the course of fish development [39]. Studies in *Danio rerio* demonstrated that the gut microbiota depends on the developmental stage of the host. At the larval stage, representatives of the phylum Proteobacteria predominated in the microbiota composition (*Vibrio*, *Aeromonas*, *Plesiomonas*, *Pseudomonas*, *Shewanella*, *Acinetobacter*). In juvenile and adult individuals, Proteobacteria and Fusobacteria co-dominated, with *Cetobacterium* and *Aeromonas* being the most significant genera [16]. A comparison of gut microbiomes between juvenile and adult Atlantic salmon *Salmo salar* revealed that juveniles exhibited greater intra-individual (alpha) diversity of the gut microbiota, while adults showed greater inter-individual (beta) diversity. Firmicutes predominated in the gut microbiomes of immature individuals, whereas Proteobacteria predominated in adult gut microbiomes [40].

#### 2.2.4. Species-Specific Characteristics and Host Genetics

The genetic characteristics of the species, developmental stage, and physiological condition also exert a significant influence on microbiome composition. A study of four herbivorous reef-fish species (*Acanthurus chirurgus*, *Kyphosus* sp., *Scarus trispinosus*, and *Sparisoma axillare*) demonstrated that despite similarities in diet, the microbiomes of these fish exhibited species-specific differences [41]. In the three-spined stickleback *Gasterosteus aculeatus*, genomic variations between populations shaped differences in the gut microbiome [14]. The gut microbiota of goldfish *C. auratus* differed between diploid and triploid individuals, with increased abundance of the genus *Vibrio* in triploids [42]. High intraspecific variability in bacterial and eukaryotic sequences in microbiomes was also demonstrated for individual Atlantic sturgeon [37]. A large-scale metagenomic analysis of 903 gut/skin samples from 121 freshwater fish species in southwestern China revealed that host phylogeny dominates microbial community variability, explaining 48.2% (skin) and 22.28% (gut) of its variance [26].

#### 2.2.5. Stress Factors

Changes in housing conditions, water quality, and stocking density can lead to alterations in microbiome composition. Nine intestinal microbial genera responsive to crowding stress were identified in the gut microbiome of blunt-snout bream (*Megalobrama amblycephala*) maintained under high stocking density. The authors demonstrated that the gut microbiome may be involved in the response to overcrowding and, accordingly, in the adaptation of fish to environmental stressors via the pentose phosphate pathway [43]. In individuals of gilthead sea bream (*Sparus aurata*, L. 1758) fed high levels of fish meal and fat, a significant decline in gut microbiota biodiversity was observed at high rearing density, possibly indicating increased social stress associated with feed competition under this regime. A reduction in the abundance of lactobacilli—an indicator of a healthy microflora—was also observed under high crowding conditions [44].

#### 2.2.6. Random (Stochastic) Processes

Alongside deterministic (selective) factors, stochastic processes play an important role. In aquaculture systems, this leads to high variability in microbiome composition even among individuals of the same species reared under identical conditions. For *Schizothorax chongi*, an endemic freshwater fish species, gut microbiota assembly was shown to be driven predominantly by stochastic processes in both wild and cultured individuals [45]. In a study of the gut microbiomes of three freshwater fish species (*Ctenopharyngodon idellus*, *Siniperca chuatsi*, and *Silurus meridionalis*), it was found that as fish developed, phylogenetic clustering of local communities and the deterministic processes governing community succession became less pronounced [46]. A comparison of the microbiomes of sea cucumber *Apostichopus japonicus* cultured in five different production systems (open pond, enclosed workshop, net cage, hanging cage, sea farm) revealed that stochastic processes predominated in the formation of microbial communities across the different systems [47].

Thus, the gut microbiome of aquatic organisms represents a complex and dynamic ecosystem composed of hundreds of microbial species that play important roles in digestion, immunity, and the overall health of the host.

## 3. Role of the Microbiome in the Immune System of Aquatic Organisms and Their Protection Against Pathogenic Bacteria

### 3.1. Interaction with the Host Immune System

The intestinal microbiome of aquatic organisms plays a fundamental role in the formation and regulation of the host immune response, acting as a critical component of both the innate and adaptive immune systems [3]. The mucosal surfaces of fish—including the intestine, skin, and gills—are in direct contact with the aquatic environment and are continuously exposed to enormous numbers of microorganisms, some of which may pose a threat to host health. Under these conditions, the microbiome fulfils key protective functions by participating in the maintenance of homeostasis between commensal microorganisms and the host immune system [48].

The interaction of the microbiome with the intestinal epithelium constitutes a complex process in which microorganisms stimulate the production of protective factors required for maintaining the barrier function of the mucosa [20]. Intestinal epithelial cells of fish produce mucins—high-molecular-weight glycoproteins that form a gel-like mucus layer covering the epithelium and serving as the first line of defense against pathogens [49]. Various types of mucins have been identified in the fish intestine, including intestinal mucin (I-Muc), mucin 2 (Muc2), mucin 13 (Muc13), mucin 18 (Muc18), and mucin 19 (Muc19). These mucins are subdivided into two main groups: membrane-bound mucins (I-Muc, Muc13, Muc18) and secreted gel-forming mucins (Muc2, Muc2-like, Muc19) [50]. Mucin gene expression is tissue-specific; Muc18 is the predominant mucin in fish skin, and its expression can be upregulated upon administration of β-glucan. The gut microbiome stimulates mucin secretion by goblet cells, thereby promoting the formation of a protective barrier that prevents the translocation of microorganisms from the intestinal lumen [51]. This mucus barrier not only physically separates the microbiota from the epithelium but also contains various antimicrobial factors, including secretory immunoglobulins and antimicrobial peptides [49].

Antimicrobial peptides (AMPs) constitute a critical component of the innate immunity of fish and are synthesized by both epithelial cells and immune cells under the influence of the microbiome [52]. Fish express all major classes of AMPs, including defensins, cathelicidins, hepcidins, histone-derived peptides, and piscidins [53]. Among the most important fish AMPs are piscidins—fish-specific peptides with a broad spectrum of antimicrobial activity that are particularly noteworthy [53]; pleurocidin, which is capable of suppressing the growth of both Gram-positive and Gram-negative bacteria by disrupting bacterial membranes [54]; and epinecidin-1—an antibacterial peptide belonging to the piscidin family that is likewise capable of disrupting bacterial membranes [55]. Certain fish AMPs, particularly those from marine species, retain activity at elevated salinity, making them sufficiently effective in the marine environment [56]. However, salt tolerance is not a universal property of all AMPs—for some peptides, high salinity reduces antimicrobial activity [57].

The intestinal microbiome of fish exerts a significant influence on the activation of pattern-recognition receptors, including Toll-like receptors (TLRs), and on cytokine production [58]. These interactions are critical for maintaining immune homeostasis and ensuring an adequate immune response to pathogens [59].

TLRs constitute a family of receptors that recognize conserved pathogen-associated structures and activate signaling cascades to regulate the immune response [51]. In the fish intestine, TLR2 and TLR5 play a particularly important role in the recognition of microbiota-associated molecular patterns (MAMPs) of probiotic bacteria. For example, studies in grouper (*Epinephelus coioides*) demonstrated that the probiotic bacterium *Psychrobacter* sp. SE6 induced elevated expression of TLR2 and TLR5, the adaptor protein MyD88, and cytokines (IL-1β, IL-8, and TGF-β1); moreover, a MyD88-independent TLR2 signaling pathway may also participate in the recognition of probiotics in fish [58]. In gnotobiotic zebrafish, depletion of the intestinal microbiome altered the TLR2–MyD88 signaling pathway, reduced the neutrophil response, and impaired type I interferon-mediated antiviral innate immunity. It was further demonstrated that the commensal bacterium *Cetobacterium somerae* could restore TLR2- and neutrophil-dependent type I interferon responses, and that exopolysaccharides of *C. somerae* (CsEPS) were the effector molecules interacting with TLR2 to mediate the type I interferon-dependent antiviral function [60].

The gut microbiome also actively participates in modulating the production of key cytokines, including interleukin-1β (IL-1β), interleukin-10 (IL-10), and tumor necrosis factor-α (TNF-α) [61,62]. These cytokines play a critical role in regulating the inflammatory response and maintaining immune homeostasis. Interleukin-10 (IL-10) is a key anti-inflammatory cytokine that suppresses the expression of pro-inflammatory cytokines [63]. In fish, IL-10 shows high expression in the liver, spleen, kidneys, and other immune organs. Studies in channel catfish demonstrated that IL-10 is markedly upregulated in response to stimulation with lipopolysaccharide, polyinosinic–polycytidylic acid, phytohemagglutinin, and phorbol ester, indicating its central role in the immune response [62]. Functional studies in Chinese perch (*S. chuatsi*) demonstrated that IL-10 effectively suppresses the expression of the pro-inflammatory cytokines IL-6, IL-1β, IL-8, and TNF-α in splenocytes stimulated with LPS [61]. This effect is mediated via IL-10R1 and IL-10R2 receptors, which activate the JAK–STAT signaling pathway and induce expression of suppressor of cytokine signaling 3 (SOCS3).

Interleukin-1β (IL-1β) and TNF-α are key pro-inflammatory cytokines whose production is closely linked to the composition and activity of the intestinal microbiome. During spring viremia of carp virus (SVCV) infection, a substantial alteration in microbiota composition is observed, with an increase in Proteobacteria and a decrease in Fusobacteria, accompanied by enhanced expression of immune and antiviral genes [48]. A probiotic, bacteriocin-producing strain, *Enterococcus thailandicus* HR23, isolated from the gut tract of large yellow croaker (*Larimichthys polyactis*), demonstrated the ability to activate immune pathways such as THαβ and TH1, while simultaneously reducing the expression level of the inflammation-associated *IL-1β* gene in rainbow trout [64].

### 3.2. Protection Against Pathogens

The intestinal microbiome of aquatic organisms provides protection against pathogenic microorganisms through various mechanisms, including competitive exclusion, the production of antimicrobial substances, and the occupation of ecological niches [51]. These mechanisms constitute a complex system of interactions that maintains microbial balance and prevents colonization by pathogens.

*Competitive exclusion*. Competitive exclusion is accomplished via two principal mechanisms: direct suppression of competitors and competition for resources [65]. The normal intestinal microbiota protects the host by depriving invading pathogens of nutrients, secreting a broad spectrum of antimicrobial substances, and occupying ecological niches. Studies in Nile tilapia (*Oreochromis niloticus*) demonstrated that a competitive exclusion culture derived from the intestinal microbiome exhibits significant antimicrobial activity against the fish pathogen *Streptococcus agalactiae* [66]. During the initial days of cultivation, the genus *Cetobacterium* was dominant, which coincided with the maximum antimicrobial effect against the pathogen. Over time, the composition of the bacterial community changed, and by day 33, *Lactococcus* spp. had become the most abundant member.

*Bacteriocin synthesis.* Bacteriocins are ribosomally synthesized antimicrobial peptides produced by bacteria to suppress the growth of closely related species [67]. In the intestinal microbiome of aquatic organisms, bacteriocins play an important role in maintaining microbial balance and protecting against pathogens. For example, Feliatra et al. showed that probiotic bacteria isolated from tiger shrimp (*Penaeus monodon*) and freshwater shrimp (*Macrobrachium rosenbergii*) are capable of producing bacteriocins with high activity against the pathogenic bacteria *Vibrio alginolyticus*, *A. hydrophila*, and *Pseudomonas stutzeri* [68]. Using mathematical modelling, the mechanism of inhibition of the pathogenic bacterium *Morganella morganii* by the strain *Lactiplantibacillus plantarum* His6 was investigated, and it was found that *Lpb. plantarum* His6 can enhance bacteriocin production while simultaneously reducing the yields of glycerophospholipids and fatty acids associated with outer membrane formation, thereby suppressing the growth of *M. morganii* YC16 [69].

*Biological mechanisms of protection.* In addition to direct antimicrobial effects, the microbiome participates in protection through biological mechanisms, including the creation of unfavorable conditions for pathogens (low oxygen levels), chemical factors (organic acids), and biochemical substances [66]. These mechanisms act synergistically, providing multi-level protection against pathogenic microorganisms.

The normal intestinal microbiome promotes the maturation and renewal of the epithelium, which in turn regulates immune responses [70]. A mature intestinal mucosa is also critically important for distinguishing pathogens from commensal microorganisms via pattern-recognition receptors, which detect bacterial antigens and activate signaling cascades to regulate immune responses [71]. Thus, the role of the microbiome in the immune system of fish is multifaceted and encompasses both direct and indirect mechanisms of host protection.

## 4. Effect of the Microbiome on the Productivity of Aquatic Organisms

The gut microbiome of aquatic organisms plays a key role in ensuring fish productivity by participating in digestion, nutrient assimilation, and maintenance of the overall health status of the host. Intestinal microbiota functions as a kind of “additional organ”, providing metabolic processes that are inaccessible to the fish organism itself [72]. A balanced microbiome promotes optimal development and functioning of the gastrointestinal tract, which is important for efficient feed utilization and, consequently, for growth and productivity in aquaculture [73].

### 4.1. Participation in Nutrient Assimilation

The gut microbiome of aquatic organisms possesses a unique set of enzymatic systems that complement the host’s digestive capabilities and allow for more efficient extraction of energy and nutrients from food [12]. Studies show that intestinal bacteria are capable of synthesizing a wide spectrum of digestive enzymes, including amylases, lipases, proteases, and cellulases, which participate in the breakdown of complex organic compounds into simpler, readily assimilable forms [74]. This additional enzymatic activity is particularly important for fish species that are unable to independently synthesize certain enzymes required for digesting the components of their natural diet [29]. For example, herbivorous fish face a particular challenge in digesting plant-based food, since their own enzymatic systems are unable to efficiently break down complex plant polysaccharides such as cellulose and hemicellulose. Cellulolytic bacteria inhabiting the intestine of herbivorous fish ensure the breakdown of these complex carbohydrates into simpler sugars that can be assimilated by the host [15]. Studies show that significantly higher diversity and abundance of cellulolytic bacteria are found in the intestine of herbivorous fish compared with predatory species. In herbivorous species, cellulolytic bacteria constituted approximately 7.47–7.63% of the total microbiome composition at the genus level; in predatory species their proportion was only 2.15–2.20%, whereas in omnivorous and planktivorous fish species it ranged from 4.19% to 5.27% [29]. Functional studies showed that in herbivorous fish such as grass carp (*C. idella*), increased consumption of dietary plant fiber leads to increased diversity of cellulolytic bacteria in the intestine, indicating a close relationship between diet composition and microbiome structure, as well as the ability of the microbiome to adapt to changes in the host’s nutrition [75]. For bacteria of the genus *Vibrio* from the intestine of blenny, the presence of alginate lyase, amylase, chitinase, and mannosidase genes in their genomes was demonstrated, indicating the role of these bacteria in the breakdown of algal polysaccharides [76].

Intestinal bacteria are capable of enhancing nutrient assimilation by the host organism, as was demonstrated using the example of the freshwater mandarin fish *S. chuatsi*. The gut microbiota of fast-growing *S. chuatsi* individuals was enriched with representatives of Fusobacteria and, at the genus level, *Cetobacterium* and *Leuconostoc*. The authors suggested that these probiotic bacteria contributed to improved nutrient assimilation by the host organism and to the efficiency of energy utilization through regulation of membrane lipid composition, stimulation of fatty acid metabolism, and synthesis of steroid hormones [77].

Metagenomic studies have revealed that the gut microbiome of fish possesses significant functional potential related to carbohydrate, protein, and lipid metabolism. Thus, using the example of fish from three different trophic levels (the herbivorous yellow tang *Zebrasoma flavescens*, the predatory Picasso triggerfish *Rhinecanthus aculeatus*, and the omnivorous arc-eye hawkfish *Paracirrhites arcatus*), a similarity in the core functions of the microbiome at the gene level was found, including glycan, protein, energy, and amino acid metabolism, but with certain differences in their representation [35]. In herbivorous and omnivorous fish, genes associated with carbohydrate metabolism—such as starch, sucrose, fructose, mannose, galactose, and glycolysis/gluconeogenesis—were more highly represented than in predatory and planktivorous species [35].

### 4.2. Synthesis of Biologically Active Substances

The gut microbiome of fish also plays an important role in the synthesis of vitamins and short-chain fatty acids (SCFAs), which cannot be produced by the host organism itself or are supplied in insufficient amounts through food [78]. These compounds are required for the normal functioning of various physiological processes, including metabolism, the immune response, and organismal development [79]. Metagenomic studies have shown that the microbial communities of the fish gut from different trophic levels possess the capacity to synthesize vitamins B1, B2, B7, B9, and B12 [35]. These vitamins play an important role in energy metabolism, DNA synthesis, nervous system function, and other key processes. According to research, vitamin B1 (thiamine) can be synthesized by various bacteria, including *Bacteroides fragilis*, *Prevotella copri*, *Lactobacillus plantarum*, *Bifidobacterium infantis*, and *B. bifidum*, whereas vitamin B2 (riboflavin) can be synthesized by *B. fragilis*, *P. copri*, and *L. plantarum* [80,81].

Vitamin B12 (cobalamin) is synthesized by specific bacteria of the fish gut microbiome, which is particularly important since this vitamin cannot be synthesized by the host and must be supplied through food or from symbiotic microorganisms. The capacity for vitamin B12 synthesis has been demonstrated for *Bacteroides* type A, *Clostridium* [82], and *C. somerae* [83].

The capacity for vitamin synthesis differs among the microbiomes of fish from different trophic groups. For example, in the above-mentioned study by Wu et al., it was shown that thiamine (B1) could be synthesized by 7 out of 18 microbial genomes in predatory fish, 10 out of 18 in herbivorous fish, and 3 out of 7 in invertebrate-feeding fish [35]. Similar differences were observed for other vitamins, indicating microbiome specialization depending on the feeding type of the host.

Short-chain fatty acids (SCFAs) are small organic acids that are produced as a result of microbial fermentation of non-digestible carbohydrates in the fish intestine [84,85], which allows for additional energy to be extracted from food. The main SCFAs produced by the intestinal microbiota are acetate, propionate, and butyrate [85]. SCFA synthesis is carried out by various groups of bacteria, including representatives of the phyla Firmicutes and Bacteroidetes [84]. It was found that the ratio of short-chain fatty acids in different sections of the intestine may vary, which is attributable to the functional compartmentalization of the microbial community of the hindgut [86].

SCFAs perform several important functions in the fish organism: they serve as an energy source, regulate intestinal pH by creating unfavorable conditions for pathogenic microorganisms, modulate the immune response and inflammatory processes, affect the permeability of the intestinal barrier, and participate in the regulation of lipid and carbohydrate metabolism [87]. A study of the effects of SCFAs on the intestinal immune status of European sea bass (using intestinal explants ex vivo) showed that butyrate and propionate influenced the expression of genes associated with immunity both before and after challenge with *Vibrio anguillarum* bacteria, but did not affect the expression of genes associated with oxidative stress [87].

When the normal composition and functions of the gut microbiome in fish are disrupted, a dysbiotic state may develop, which is associated with growth retardation and increased mortality [6]. Studies show that microbiome dysbiosis is associated with various pathological processes, including disorders of digestion, immune function, and metabolism [88]. For example, a study in zebrafish demonstrated that chronic exposure to antimicrobials caused significant changes in the composition of the gut microbiome and increased susceptibility to pathogens [6], including bacteria such as *Enterococcus*, *Stenotrophomonas*, *Aeromonas*, and *Raoultella* [89].

Thus, the gut microbiome plays a fundamental role in ensuring fish productivity by participating in nutrient assimilation, synthesis of vitamins and SCFAs, and maintaining intestinal health and immune function.

## 5. Practical Aspects of Microbiome Modulation

Modulation of the gut microbiome of aquatic organisms represents a promising direction in modern aquaculture, enabling improvements in productivity, disease resistance, and the overall health of cultured species. The practical application of various biologically active supplements—including probiotics, prebiotics, and their combinations (synbiotics)—opens new opportunities for the sustainable development of fish farming without the use of antimicrobials and other chemotherapeutic agents.

### 5.1. Probiotics

Probiotics are live microorganisms that, when administered in adequate amounts, confer a beneficial effect on the health of the host [90]. In aquaculture, the most widely used probiotic bacteria belong to the genera *Bacillus*, *Bifidobacterium*, lactic acid bacteria *Lactobacillus*, *Enterococcus*, and *Streptococcus* [91], as well as certain yeast species such as *Saccharomyces* [92], which demonstrate high efficacy in improving growth, immunity, and resistance to pathogens in various fish species.

Bacteria of the genus *Vibrio* are among the most prevalent pathogens in aquaculture, causing significant economic losses in fish-farming enterprises [93]. Probiotic strains *B. subtilis* and *L. plantarum* in the study by Touraki et al. [94] demonstrated high efficacy in suppressing the growth of the European seabass-pathogenic strain *V. anguillarum* through the production of antibacterial substances. Strains of *B. subtilis* and *B. licheniformis* isolated from fish intestines and marine sediments exhibit pronounced antagonistic activity against *V. harveyi* and *V. anguillarum*, the principal causative agents of vibriosis in marine fish [93]. Studies have shown that these probiotic bacteria are capable of producing bacteriocins—ribosomally synthesized antimicrobial peptides—that effectively suppress the growth of pathogenic vibrios [93]. When Nile tilapia were fed a probiotic supplement containing the bacteriocin-producing strain *Lactococcus lactis* subsp. *lactis* A7, a significant improvement in host immune parameters was observed, including reduced serum ALT levels and elevated total protein, lysozyme activity, and lymphocyte counts [95]. In addition, increased digestive enzyme activity and a reduced abundance of aeromonads in the intestinal microbiota were recorded. The probiotic strain *B. subtilis* subsp. *inaquosorum* BSXE-2102, applied in shrimp aquaculture, demonstrated potent antagonistic activity against eight aquatic pathogenic strains (predominantly *Vibrio*), and genomic analysis revealed multiple genes responsible for the biosynthesis of 12 secondary metabolites, including surfactin, iturin A, bacillomycin D (an iturin variant), mycosubtilin (an iturin variant), fengycin, and others [96]. Strain *B. subtilis* S17, isolated from sardine intestine, demonstrated strong antibacterial activity against *V. harveyi* and *V. anguillarum*, as well as high resistance to gastrointestinal tract conditions and the ability to adhere to fish mucus [93].

Lactic acid bacteria of the genus *Lactobacillus* are also effective in suppressing *Vibrio* through various mechanisms, including the production of organic acids (lactic, acetic) that lower the environmental pH and create unfavorable conditions for pathogen growth [97]; competition for nutrients and adhesion receptors on the intestinal epithelium, thereby preventing pathogen colonization [98]; production of bacteriocins and other antimicrobial compounds that act specifically on pathogenic bacteria [99]; and suppression of virulence gene expression in pathogens via quorum-sensing mechanisms [99].

In vivo studies confirm the efficacy of probiotics in protection against vibriosis. For example, application of *Paenibacillus* spp. and *B. cereus* at a concentration of 10^4^–10^5^ CFU mL^−1^ significantly reduced mortality in postlarvae of the shrimp *P. monodon* upon challenge with *V. harveyi* [100]. The probiotic SYNLAC Prime, comprising strains *Enterococcus faecium* EF08, *Pediococcus pentosaceus* PP49, *Lactobacillus plantarum* LP28, *Lactobacillus acidophilus* LA1063, and *B. subtilis*, modulated the gut microbiota of Pacific white shrimp *P. vannamei*, reducing *Vibrio* abundance [101]. Earlier studies also showed that dietary supplementation with a multi-strain probiotic formula (SYNSEA) containing *Lactobacillus* and *Bacillus* over a 56-day feeding trial effectively suppressed *Vibrio*-like populations in the gut of white shrimp [102]. Similarly, the probiotic strain *Vagococcus fluvialis* provided effective protection of European seabass (*Dicentrarchus labrax*) against *V. anguillarum* infection [103]. The probiotic strategies used in crustaceans differ in several respects from those applied in fish; these differences are compared in Section 5.4.

Probiotics exert a significant influence on the immune system of aquatic organisms, modulating the expression of key immune genes, including immunoglobulin and cytokine genes [104,105]. Of particular interest is the influence of probiotics on the expression of IgT (immunoglobulin T)—a specialized immunoglobulin that plays a central role in mucosal immunity in fish—and IL-10 (interleukin-10), an important anti-inflammatory cytokine [106,107]. IgT is the functional analogue of mammalian IgA and plays a key role in protecting fish mucosal surfaces from pathogens [106]. Studies indicate that probiotics are capable of stimulating IgT gene expression and increasing the number of IgT+ B cells in the mucosal surfaces of the intestine, gills, and skin of fish [108].

In rainbow trout (*Oncorhynchus mykiss*) infected with *Flavobacterium columnare*, a significant increase in IgT expression was observed in the gills on days 28 and 75 post-infection, accompanied by accumulation of IgT+ B cells and a strong bacterium-specific IgT response in the gill lamellae [108]. The application of probiotics, particularly *B. subtilis*, contributed to the augmentation of this protective response. Studies have shown that certain probiotic strains can induce IgT production in fish mucosal surfaces as early as 4 days after immunization [105]. This effect was enhanced upon re-administration of the probiotic 30 days after the initial immunization.

IL-10 is a key anti-inflammatory cytokine that suppresses the expression of pro-inflammatory cytokines and plays an important role in regulating the immune response [109]. Probiotics are capable of modulating IL-10 expression in various fish tissues, thereby contributing to the maintenance of immune homeostasis [109]. Studies on the yellow catfish (*Pelteobagrus fulvidraco*) showed that the probiotic strain *Clostridium butyricum* significantly elevated IL-10 expression in the intestine of the fish [110]. Moreover, following challenge with the pathogen *Aeromonas punctata*, IL-10 expression in the intestine of fish that received the probiotic was even higher, indicating an anti-inflammatory role of the probiotic [110]. Interestingly, a simultaneous reduction in the expression of the pro-inflammatory cytokine IL-8 was observed, which may indicate a suppressive role of IL-10 with respect to IL-8 transcription. In another study, it was shown that IL-10 gene expression levels in the intestine of fish receiving probiotics (designated Pro-A, Pro-B, Pro-C) and oxytetracycline (OTC) were significantly higher than in the control group [109]. This suggests that probiotics may attenuate inflammation in fish through upregulation of cytokine gene expression (IL-1β and IL-10).

The probiotic *B. subtilis* also demonstrated the ability to enhance the systemic specific IgM response to vaccination in rainbow trout (*O. mykiss*) [111]. Fish that received the probiotic for 30 days prior to immunization with various antigens, including a *Yersinia ruckeri* bacterin and a DNA vaccine against viral hemorrhagic septicemia virus, exhibited higher titers of specific IgM antibodies and increased antibody affinities for the antigens.

### 5.2. Prebiotics: Oligosaccharides as Stimulants of Beneficial Bacterial Growth

Prebiotics are non-digestible dietary components that selectively stimulate the growth and/or activity of certain groups of microorganisms in the gut, thereby contributing to improvements in host health [112]. In aquaculture, the most widely used prebiotics are oligosaccharide-based compounds, which have demonstrated high efficacy in stimulating the growth of beneficial bacteria in the fish intestine [113]. Oligosaccharides are short sugar chains that are not digested by enzymes in the upper gastrointestinal tract of fish, but can be fermented by certain bacterial groups in the intestine. The most studied and effective prebiotics for fish are xylooligosaccharides, galactooligosaccharides, fructooligosaccharides, and mannanoligosaccharides. Xylooligosaccharides (XOS) are products of xylan hydrolysis, consisting of xylose chains of varying length. XOS are widely applied in aquaculture owing to their ability to selectively stimulate the growth of beneficial bacteria (e.g., Bacteroidetes, Actinobacteria), enhance immune responses, and improve growth performance [114]. Bai et al. [115] demonstrated that XOS supplementation elevated IgM, C3, and LZM activity levels in yellow catfish. Studies in tilapia (*O. niloticus*) showed that the addition of XOS to the diet significantly reduced the feed conversion ratio (FCR) and improved fish growth [112]. In 30-day-old tilapia fry, XOS reduced FCR by 34.4% compared with the control group. XOS in the diet of Nile tilapia fed a high-carbohydrate diet promoted increased growth rates and reduced fat accumulation, which was likely associated with the intestinal microbiota [116]. However, XOS did not exert a significant effect on growth rates in fry of the Caspian white fish *Rutilus frisii kutum* following dietary addition at 1%, 2%, and 3% XOS [117].

Galactooligosaccharides (GOS)—oligosaccharides composed of galactose with a terminal glucose residue—also demonstrated the ability to reduce FCR in tilapia by 34.4% in 30-day-old fry and by 11.9% in 90-day-old fish [112]. Fructooligosaccharides (FOS) and inulin are oligosaccharides composed of fructose with a terminal glucose residue. FOS with shorter chains (DP ≤ 10) can be fermented by a larger number of microorganisms, whereas inulin with longer chains (DP ≤ 60) is metabolized by only a few bacterial species.

Mannanoligosaccharides (MOS) are derivatives of the cell walls of the yeast *Saccharomyces cerevisiae*. These oligosaccharides primarily contain mannose as the main carbohydrate component. MOS are capable of binding to pathogenic bacteria, thereby preventing their adhesion to the intestinal epithelium. The addition of concentrated mannan oligosaccharide (cMOS) to the diet of grass carp (*C. idellus*) contributed to improved growth performance and immunity, as well as survival following challenge with *Ichthyophthirius multifiliis* [118]. Application of MOS improved the growth and survival of pompano (*Trachinotus ovatus*), increased total aerobic bacterial counts, reduced intestinal vibrio counts, and improved feed and nutrient digestibility. The effect of the supplementation was dose-dependent; the optimal dose was 0.4%. Higher concentrations had no effect on growth performance and survival [119]. Addition of MOS at 1.5 g per kg of diet in hybrid red tilapia (*O. niloticus* × *O. mossambicus*) contributed to improved reproductive function and elevated expression of reproductive genes, while 1 g MOS per kg feed improved hematological and biochemical parameters. No significant effects were observed at a dose of 0.5 g kg^−1^ MOS [120]. Feeding milkfish (*Chanos chanos*) a diet containing 3 g kg^−1^ MOS yielded the best growth performance, digestive antioxidant protection, immune response, and disease resistance. The lowest dose (1 g kg^−1^) affected only fish growth and the activity of the antioxidant enzymes superoxide dismutase and catalase [121].

It should be noted that a number of studies reported an absence of significant MOS effects on the health of aquaculture species. For example, the addition of MOS and inulin did not result in a substantial change in growth performance or survival of African catfish larvae compared with the control group. Despite differences in MOS dosage, the prebiotics had a lesser effect on growth parameters, and their action may have been indirect [122]. Adeoye et al. [123] also reported that the addition of 0.2% MOS had no significant effect on growth parameters such as mean final weight and mean daily body weight gain in hybrid catfish.

In addition to the oligosaccharides described above, chitosan oligosaccharides (COS) and alginate oligosaccharides (AOS) are also applied as prebiotic supplements for aquaculture species. Addition of 0.3% chitosan oligosaccharides (COS) to the diet of the predatory fish *S. chuatsi* improved growth performance by 71% and also altered the intestinal microbiota, increasing the proportion of *Cetobacterium* to 54.2% and suppressing the growth of 19 pathogenic genera, including *Klebsiella* and *Pseudomonas*. In addition, activation of the hepatic MAPK-NF-κB signaling pathway along the gut–liver axis, support of immune cell survival under inflammatory conditions, and elevated levels of intestinal immune substances were demonstrated [124]. COS significantly enhanced resistance of grass carp *C. idella* to grass carp reovirus GCRV-II by stimulating secretion of natural immunoglobulin M [125].

In Chinese seabass (*Lateolabrax maculatus*), dietary AOS supplementation improved growth performance, lipid metabolism, antioxidant capacity, and immunity [126]. Dietary addition of AOS to red swamp crayfish (*Procambarus clarkii*) enhanced antioxidant defense and immune function in *P. clarkii*, thereby reducing nitrite-induced gill damage and immunosuppression, primarily through modulation of the NFIL3/NF-κB signaling axis [127]. AOS at 0.5–1.0% promoted accelerated growth in shrimp *L. vannamei*, increased digestive enzyme activity, and elevated activities of enzymes associated with immunity and antioxidant defense [128]. In juvenile largemouth bass (*Micropterus salmoides*), AOS supplementation increased superoxide dismutase, catalase, and glutathione peroxidase activity in the liver and intestine, improved liver and intestinal morphology, and regulated intestinal microbiota by reducing the abundance of pathogenic microorganisms. In addition, AOS enhanced immunity and resistance to *A. hydrophila* challenge, but did not affect fish growth performance [129].

The mechanisms of action of prebiotics in the fish intestine may be mediated by selective stimulation of beneficial bacterial growth—oligosaccharides serve as a substrate for the growth of certain bacterial groups such as *Lactobacillus*, *Bifidobacterium*, and certain *Bacillus* species. For example, application of XOS and GOS in tilapia led to increased abundance of *C. ruminantium*, *Brevinema andersonii*, *Shewanella amazonensis*, *Reyranella massiliensis*, and *Chitinilyticum aquatile* in the intestine [112]. Furthermore, as a result of prebiotic fermentation by beneficial bacteria, short-chain fatty acids (SCFAs) (acetate, propionate, butyrate) are produced, which lower intestinal pH, creating unfavorable conditions for pathogens, and serve as an energy source for intestinal epithelial cells. Prebiotics can enhance the non-specific immune response in fish by increasing lysozyme activity, complement activity, and phagocytosis. It was also found that prebiotics contribute to increased villus height and crypt depth in the intestine, resulting in an expanded surface area for nutrient absorption [130].

The efficacy of prebiotic use depends on the age of the fish, their species, and rearing conditions. Studies indicate that prebiotics are more effective when applied to younger fish, and that a combination of several oligosaccharide types can produce a more pronounced effect. For example, the combination of XOS and GOS in 90-day-old tilapia reduced FCR by 20.2% compared with the control group, which exceeded the effect of each prebiotic applied individually (11.9%) [112]. A synergistic effect of mannanoligosaccharides and vitamin E (VE) was noted—pompano receiving 0.4% MOS and 0.01% VE showed improved growth, survival, feed digestibility, and stress resistance. In the intestine, the number of aerobic bacteria increased, while the *Vibrio* count was significantly reduced [131]. Pompano fed a mixture of 0.1% β-glucan (BG) and 0.4% mannanoligosaccharides (MOS) demonstrated higher growth rates, lower coefficient of variation (CV), improved hematological parameters, feed and nutrient assimilation, and intestinal morphology [132]. A detailed analysis of the effects of prebiotic mixtures on various aspects of aquaculture species health is presented in the work of Wee et al. [133].

An important factor influencing the efficacy of prebiotic use is their dietary dosage. Zhang et al. [134] demonstrated in a study on juvenile grass carp (*C. idellus*) that fish receiving 0.05%, 0.1%, and 0.2% XOS in the diet had significantly higher body weight, resting body weight, and specific growth rate than fish receiving 0.6% XOS or the control group. This may be attributable to the fact that an excess of prebiotics can be harmful to fish and rapidly leads to increased osmotic pressure in the intestine, which may cause diarrhea and nutrient loss.

Thus, prebiotics are an effective means of improving aquaculture productivity; however, their effects are strongly dependent on numerous factors, including the species, age, and husbandry conditions of the aquatic organism, as well as the dietary dosage.

### 5.3. Synbiotics: Combined Preparations for Enhanced Efficacy

Synbiotics represent a combination of probiotics and prebiotics that provides a synergistic effect exceeding the action of each component individually [135]. The concept of synbiotic application is based on the idea that prebiotics can selectively stimulate the growth and activity of probiotic bacteria, thereby amplifying their beneficial influence on the host organism. In aquaculture, synbiotics have been used for more than 10 years and have demonstrated high efficacy in improving growth, immunity, and disease resistance in various aquatic organisms [136]. The action of synbiotics involves several mechanisms. First, prebiotics serve as a selective substrate for the growth of probiotic bacteria, which promotes their better survival and colonization in the fish intestine [137]. This is especially important in open aquaculture systems, where maintaining a stable probiotic population may be challenging. Second, synbiotics stimulate the production of various bioactive compounds by probiotic bacteria, including enzymes, organic acids, bacteriocins, and other antimicrobial substances [138]. These compounds can suppress pathogen growth, improve digestion, and modulate the host immune response. Synbiotics can exert a complex influence on the immune system, activating both innate and adaptive immunity in fish, and stimulating the production of cytokines, as well as the activity of phagocytes, lysozyme, and complement [139]. Additionally, synbiotic preparations improve digestion and nutrient assimilation in aquatic organisms by stimulating digestive enzyme activity and improving intestinal morphology. Studies on the Pacific white shrimp *L. vannamei* showed that the application of a synbiotic composed of the probiotic strain *Pseudoalteromonas piscicida* 1Ub in combination with the prebiotic fructooligosaccharide (FOS) had a positive effect on growth performance and immune responses in Pacific white shrimp, and reduced shrimp mortality following co-infection with white spot syndrome virus (WSSV) and *V. harveyi* [140]. When larvae of hybrid catfish were fed live Artemia enriched with synbiotics (*Enterococcus faecium* and *Enterococcus faecalis* + inulin), improvements in larval growth performance and intestinal health were demonstrated [141].

Antioxidant activity of synbiotics in fish has been demonstrated, as expressed by increased levels of antioxidant enzymes such as superoxide dismutase. Specifically, application of a synbiotic comprising *Lactobacillus helveticus* (LH) and gum arabic in common carp (*Cyprinus carpio*) over 8 weeks led to improvements not only in growth performance, leukocyte counts, total serum and skin mucus immunoglobulin levels, but also in superoxide dismutase and catalase activity [138].

The efficacy of synbiotics depends on the correct selection of the probiotic–prebiotic combination. The most effective combinations are those in which the prebiotic selectively stimulates the growth of the probiotic used [135]. For example, the combination of fructooligosaccharides with *Lactobacillus*, *Bifidobacterium*, or *Bacillus* demonstrates high efficacy owing to the specificity of these prebiotics for the corresponding probiotic bacteria [62,138,142].

Synbiotics may be particularly effective under stress conditions or at high fish stocking densities [143]. In European seabass, the application of a synbiotic based on mannanoligosaccharides and *P. acidilactici* contributed to the formation of a healthier microbial environment in the intestine, enhancing protection against *V. anguillarum* and improving the stress response under low-quality diets [143]. In Table 2, a list of probiotic microorganisms most widely used in aquaculture, both individually and as components of synbiotics, is presented.

### 5.4. Comparative Aspects: Fish Versus Crustaceans

Although fish and crustaceans are frequently grouped together as “aquatic organisms,” they differ substantially in immune architecture, in the spectrum of probiotics applied, and in the practical consequences of microbiome modulation; these differences are summarized in Table 3.

The most fundamental difference concerns host immunity. Fish possess both innate and adaptive immune systems, including immunoglobulin-based mucosal responses (notably IgT) and cytokine networks that probiotics can measurably modulate (Section 3.1). Crustaceans, as invertebrates, lack a vertebrate-type adaptive immune system and immunoglobulins, and defend themselves through innate mechanisms: cellular responses mediated by hemocytes (phagocytosis, encapsulation, nodule formation) and humoral effectors such as the prophenoloxidase (proPO)–melanization cascade, antimicrobial peptides (AMPs), clotting proteins, and pattern-recognition receptors, complemented by RNAi-based antiviral defense [165,166]. A limited, non-specific form of “immune priming” has also been described in shrimp [165]. As a result, the endpoints used to assess probiotic efficacy differ between the two groups: immunoglobulin (IgT/IgM) titers and cytokine expression are informative in fish, whereas phenoloxidase/proPO activity, total hemocyte counts, AMP expression, and pathogen (particularly *Vibrio*) load are the more relevant readouts in crustaceans.

These immunological differences are mirrored in the disease context and in the probiotic spectrum. In crustacean (mainly shrimp) aquaculture, the dominant threats are *V. parahaemolyticus*—the causative agent of acute hepatopancreatic necrosis disease (AHPND)—other vibrios such as *V. harveyi*, and white spot syndrome virus (WSSV), often as co-infections [4,140]. Accordingly, the probiotics reviewed here for crustaceans emphasize *Vibrio* antagonism and are drawn mainly from the genera *Bacillus*, *Lactobacillus*, *Pseudoalteromonas*, *Leuconostoc*, and *Streptococcus* (*S. phocae*): for example, *B. subtilis* subsp. *inaquosorum* BSXE-2102 suppressed eight predominantly *Vibrio* pathogens in shrimp [96]; a *Pseudoalteromonas piscicida*–fructooligosaccharide synbiotic reduced *L. vannamei* mortality after WSSV/*V. harveyi* co-infection [140]; *Leuconostoc mesenteroides* improved resistance of *P. vannamei* to *V. parahaemolyticus* [159]; and bacteriocin-producing isolates from *P. monodon* and *M. rosenbergii* inhibited *V. alginolyticus*, *A. hydrophila*, and *P. stutzeri* [68]. In fish, by contrast, a broader range of lactic acid bacteria (*Lactococcus*, *Enterococcus*, *Carnobacterium*, *Pediococcus*) and yeasts is used, and the targets extend from *Vibrio* to *Aeromonas*, *Flavobacterium*, and *Streptococcus*, with outcomes commonly reported as improved mucosal immunity, cytokine balance, and gut morphology (Section 3 and Section 5.1).

Finally, the routes and dynamics of microbiome assembly differ in ways that shape the modulation strategy. In shrimp, water and bottom sediments are a major inoculum for the gut community—contributing up to ~85% of colonizers at early developmental stages [31]—and the microbiome is further reshaped by molting, making environmental management and larval-stage intervention particularly important. In fish, the microbiome likewise forms from water and feed but becomes increasingly host-specific with maturation (Section 2.2). Overall, although probiotics, prebiotics, and synbiotics are beneficial in both groups, effective modulation requires host-specific strain selection, delivery, and efficacy criteria, and results obtained in fish should not be extrapolated uncritically to crustaceans, or vice versa.

Thus, the practical aspects of gut microbiome modulation in fish and crustaceans include the use of probiotics, prebiotics, and their combinations (synbiotics) to improve health, growth, and disease resistance in aquaculture species. The use of these biologically active supplements represents a promising alternative to antimicrobials and other chemotherapeutic agents, contributing to the sustainable development of fish farming and improvement of product quality.

## 6. Challenges and Prospects

### 6.1. Instability of Probiotic Strain Colonization in Open-System Conditions

The use of probiotics in aquaculture faces serious challenges, particularly in open rearing systems, where control over the microbial community is substantially more difficult. Instability in colonization by probiotic microorganisms represents one of the key problems limiting the efficacy of probiotic preparations in commercial fish farming [167].

In general, the stability of probiotic colonization in the fish intestine depends on a multitude of factors, including environmental conditions, the physiological characteristics of the host, and the properties of the probiotic strains themselves [168]. In open aquaculture systems, these factors acquire particular significance. For example, in a study of intestinal colonization in cod by probiotic strains, it was found that the introduced bacteria were unable to prevent the colonization of eggs by the microbiota naturally present in the incubator [167]. This indicates the complexity of managing microbial communities under open-system conditions. Studies on salmonids demonstrated that lactic acid bacteria, which are not dominant members of the normal intestinal microbiota of fish, caused only a transient change in the composition of the intestinal microbial community when administered at high doses. Within several days after the cessation of administration, a sharp decline in the abundance of the added strains was observed, and they disappeared from the gastrointestinal tract of most fish [167].

The surrounding environment exerts considerable influence—in open water bodies, continuous water exchange leads to the dilution of probiotic concentrations, thereby reducing the likelihood of their successful colonization of the fish intestine. Studies show that even with regular introduction of probiotics into the water, their concentration rapidly decreases due to currents, sedimentation, and other physical processes [168]. Competition with the autochthonous microbiota can also modulate the effect of a probiotic, since under natural conditions the fish intestine is already populated by a stable microbial community adapted to that ecological niche. Probiotic strains are therefore forced to compete with these microorganisms for nutrients and adhesion sites [169]. In addition, stochastic events play a significant role in intestinal colonization, particularly in open systems where environmental microbial diversity is high.

Several strategies may be proposed to overcome the problem of unstable probiotic colonization in open aquaculture systems. First, regular application of probiotics is necessary. Maintaining artificial dominance of probiotic strains requires their regular introduction into the rearing environment or fish feed. The use of autochthonous probiotics may also be beneficial, as employing probiotic strains isolated from the intestine of the same fish species can increase the probability of successful colonization owing to their better adaptation to the conditions of that ecological niche. Protection of probiotic cells through microencapsulation can increase their survival during passage through the stomach and promote more effective intestinal colonization. Furthermore, the combined application of probiotics with prebiotics (i.e., synbiotics, as discussed above) can enhance the survival and colonization capacity of probiotic strains.

### 6.2. Development of Personalized Feed Additives Based on Metagenomic Data

Advances in metagenomic analysis technologies are opening new opportunities for the creation of personalized feed additives tailored to the specific characteristics of the microbiome of particular aquaculture species or even individual populations thereof.

Metagenomic analysis provides a comprehensive view of the composition and functional potential of the fish gut microbiome without the need to culture microorganisms [170]. Taxonomic analysis allows the species composition of the gut microbiome to be determined for various fish species under different rearing conditions. As noted previously, the fish microbiome is characterized by relatively low diversity compared with that of mammals, with several bacterial genomes frequently accounting for more than 75% of total microbial DNA reads [171]. Functional analysis can be used to examine the metabolic pathways and functional genes of the microbiome associated with digestion, immunity, pathogen defense, and other physiological processes [172]. By comparing the taxonomic and functional microbiome profiles of fish of different species, ages, and rearing conditions, it becomes possible to identify key taxa and functional groups characteristic of healthy fish with high growth performance [171,172]. This will allow for targeted intervention on key taxa, identifying and stimulating the growth of bacterial groups associated with improved growth and health indicators in fish. Modulation of functional pathways is also feasible through the development of additives aimed at stimulating specific metabolic pathways in the microbiome that are linked to improved nutrient assimilation or the production of biologically active compounds.

A more complete understanding of the interaction between the aquatic organism host and its microbiome can be achieved by integrating metagenomics with other omics approaches (metatranscriptomics, metaproteomics, and metabolomics). Such a multi-omics approach will make it possible, for example, to reveal the functional effects of probiotics and synbiotics on microbiome-associated functions in relation to fish growth performance [173]. The development of bioinformatic tools will enable the creation of specialized databases and algorithms for the analysis of metagenomic data from aquaculture. Despite significant progress in the development of metagenomic databases for marine environments, freshwater environments remain insufficiently studied, necessitating the development of specialized tools.

The application of artificial intelligence currently stands at the forefront of science; machine learning methods can be applied to identify complex relationships between microbiome composition, environmental parameters, and fish growth and health indicators [174]. This will enable the development of more effective, personalized feed additives. However, to validate results obtained under laboratory conditions, large-scale field trials of personalized feed additives in diverse rearing environments are still necessary to evaluate their efficacy and safety.

Thus, the future of aquaculture is closely linked to the development of microbiome-oriented technologies that will enable the creation of more sustainable, productive, and environmentally sound production systems. Understanding and harnessing the potential of the microbiome will contribute not only to increased aquaculture efficiency but also to the conservation of biodiversity in aquatic ecosystems and the ensuring of food security on a global scale.

## 7. Conclusions

Research on the gut microbiome of aquatic organisms has reframed the gut microbiome of aquatic organisms from an incidental microbial assemblage into a functionally active “accessory organ” that can be deliberately managed to improve digestion, immunity, pathogen resistance, and productivity. Microbiome status in this paradigm is not only a descriptor of host condition but a controllable lever in aquaculture, provided that intervention is matched to the host, the rearing system, and the dose.

The gut microbiome of aquatic organisms is dominated by the phyla Proteobacteria, Firmicutes, Bacteroidetes, Fusobacteria, Actinobacteria, and Verrucomicrobia, the proportions of which differ between marine and freshwater species and depend on host species, age, diet, environmental factors, and stochastic processes. The microbial community begins to form during early developmental stages under the influence of water and feed, gradually stabilizes as the organism matures, and dynamically reorganizes in response to changes in rearing conditions and stressors.

The gut microbiome plays a key role in protecting aquatic organisms from pathogens, achieving this through competitive exclusion, the synthesis of bacteriocins and other antimicrobial compounds, and the establishment of biochemical conditions unfavorable to pathogens. In addition to direct antimicrobial activity, commensal bacteria modulate both innate and adaptive immunity by activating signaling through pattern recognition receptors (including Toll-like receptors), regulating the production of cytokines, lysozyme, and antimicrobial peptides, and promoting maturation of the intestinal epithelial barrier.

Microbial communities make a substantial contribution to the productivity of aquatic organisms: they expand the host’s enzymatic capabilities through amylases, proteases, lipases, and cellulases; provide the synthesis of vitamins (primarily B-group vitamins and vitamin K) and short-chain fatty acids; regulate the expression of growth-related genes; and improve the feed conversion ratio.

Fish and crustaceans, however, require distinct strategies and distinct efficacy endpoints, and results should not be extrapolated across the two groups. In fish, probiotics can be evaluated through their capacity to enhance adaptive mucosal immunity (IgT responses, immunoglobulin and cytokine regulation) alongside innate defenses. In crustaceans, which lack an immunoglobulin-based adaptive system and rely on innate mechanisms, competitive exclusion, antimicrobial peptides, and, above all, suppression of pathogens such as *Vibrio* are the decisive readouts. Probiotic panels, delivery routes, and outcome measures should be designed accordingly.

Targeted modulation of the microbiome using probiotics, prebiotics, and their combinations (synbiotics) is today one of the most promising directions in practical aquaculture. The data on probiotic strains of the genera *Bacillus*, *Lactobacillus*, *Lactococcus*, *Pediococcus*, *Enterococcus*, *Carnobacterium*, *Streptococcus*, and *Pseudomonas*, as well as the yeast *Saccharomyces*, systematized in the present review, confirm their probiotic potential across a wide range of species—from cyprinids and tilapia to salmonids, percids, and shrimp. Prebiotic effects reviewed here are consistently dose-dependent and frequently non-monotonic: intermediate doses improve growth and immunity, while excess can cause negative effects. The implication is that species-, life-stage-, and dose-specific protocols are required for reproducible results, and that reporting optimal dose windows should become standard practice. Synbiotics, owing to the synergistic action of the probiotic and the selective substrate for its growth, demonstrate particular efficacy under stress conditions and at high stocking densities.

Despite the progress achieved, a number of challenges remain unresolved. The principal challenges include unstable colonization of probiotics in open aquaculture systems, inter-species and individual variability of the microbiome, the absence of standardized protocols for synbiotic application, and insufficient knowledge of freshwater microbial communities.

Promising directions include identifying four concrete priorities: (i) multi-omics-guided strain and pathway selection, integrating metagenomics with metatranscriptomics, metaproteomics, and metabolomics to move from taxonomic description to function-targeted intervention; (ii) machine-learning models linking microbiome composition, environmental parameters, and performance to predict probiotic and synbiotic effects; (iii) large-scale field trials under commercial conditions to confirm laboratory findings; and (iv) the development of freshwater-specific microbial reference databases, which remain markedly underdeveloped relative to marine resources.

Taken together, these inferences support a clear direction of travel: microbiome-oriented approaches—from targeted modulation of the resident microbiota to the engineering of personalized, host- and system-matched synbiotic preparations—offer one of the most viable routes to antibiotic-reduced, disease-resilient, and sustainable aquaculture. Realizing this potential will depend on integrating microbiology, immunology, molecular biology, bioinformatics, and ecology and on adopting the reporting standards needed to make results comparable and field-ready.

## Figures and Tables

**Table 2:** Principal probiotic microorganisms used in aquaculture.

Bacterial Group	Species	Host	Beneficial Effects	References
Gram-positive spore-forming *Bacillus* (Firmicutes)	*B. amyloliquefaciens*	Nile tilapia (*O. niloticus*)	Positive effects on the digestive system, immunity, and microflora composition	[144,145]
	*B. cereus*	Coral grouper (*Plectropomus leopardus*)	Growth, intestinal protease and lipase activity	[146]
	*B. pumilus*	Orange-spotted grouper (*Epinephelus coioides*)	Activation of intestinal mucosal immunity; elevated expression of the antibacterial peptide epinecidin-1	[147]
	*B. subtilis*	Goldfish (*C. auratus)*	Restoration of microbiota dysbiosis caused by a high-soy diet	[112]
	*B.velezensis*	European seabass (*Dicentrarchus labrax*)	Serum bactericidal activity; survival following *Vibrio* challenge	[148]
Gram-positive lactic acid bacteria (LAB)	*Carnobacterium divergens*	Atlantic cod (*Gadus morhua*) larvae	Improved growth, survival, resistance to *V. anguillarum*	[149]
	*C. butyricum*	Large yellow croaker (*Larimichthys crocea*) larvae	Improved growth performance, intestinal development, immune response, and intestinal microbiota	[150]
	*E. lactis*	Largemouth bass (*Micropterus salmoides*)	Production of γ-aminobutyric acid; improved growth performance, intestinal development, immune response, and intestinal microbiota	[151]
	*L. lactis*	Nile tilapia (*O. niloticus*)	Improved immune parameters; enhanced digestive enzyme activity; reduced intestinal aeromonad counts; antagonism against *Streptococcus agalactiae*	[95]
	*L. acidophilus*	Nile tilapia (*O. niloticus*)	Improved growth and intestinal topography; antioxidant protection	[152]
	*L. fermentum*	Common carp (*C. carpio*)	Improved growth, hematological parameters; reinforcement of non-specific immunity	[153]
	*L. lactis*	Flounder (*Paralichthys olivaceus*)	Improved feed conversion and specific growth rate; protection of the host against streptococcosis via competitive exclusion and enhancement of innate immune responses	[154]
	*L. plantarum*	Nile tilapia (*O. niloticus*)	Improved growth performance and immunity; protection of the host against *S. agalactiae*	[155]
	*L. rhamnosus*	Nile tilapia (*O. niloticus*)	Positive effect on intestinal structure and mucosal immunity	[156]
	*Pediococcus acidilactici*	Largemouth bass (*M. salmoides*)	Improved immune function and intestinal health; resistance to *A. hydrophila* and other aquatic pathogens	[157]
	*Weissella cibaria*	Goldfish (*C. auratus*)	Enhanced immunity; survival following *A. veronii* challenge	[158]
Other Gram-positive bacteria	*Leuconostoc mesenteroides*	Shrimp (*P. vannamei*)	Improved immune regulation; resistance to *V. parahaemolyticus*	[159]
	*Streptococcus phocae*	Shrimp (*P. monodon*)	Improved growth; high larval survival upon *V. harveyi* challenge	[160]
Gram-negative bacteria	*A. veronii*	Common carp (*C. carpio*)	Improved growth performance; elevated innate immunity; survival under *A. hydrophila* infection	[73]
	*Alcaligenes faecalis*	Asian mahseer (*Tor tambroides*)	Improved microbiota composition; increased intestinal villus length, width, and surface area; SCFA production	[161]
	*Pseudoalteromonas* spp.	European seabass (*D. labrax*)	Survival following *V. harveyi* infection; anti-biofilm effect	[162]
	*V. lentus*	European seabass (*D. labrax*) larvae	Reduction in cortisol (a marker of acute stress) in blood plasma	[163]
Yeasts	*Saccharomyces cerevisiae*	Striped catfish (*Pangasianodon hypophthalmus*)	Growth and feed assimilation; enhanced immunity; increased intestinal villus height	[164]

**Table 3:** Comparison of key features relevant to gut microbiome modulation in fish and crustaceans.

Feature	Fish	Crustaceans (Mainly Shrimp)
Adaptive immunity	Present; immunoglobulins (IgM, mucosal IgT) and cytokine-mediated responses [3,105,107]	Absent; innate immunity only, with limited non-specific “immune priming” [165]
Principal immune effectors	TLR/PRR signaling, cytokines (IL-1β, TNF-α, IL-10), lysozyme, complement, mucosal Ig [3,60,61]	Hemocytes; prophenoloxidase (proPO)/melanization; antimicrobial peptides; clotting; RNAi antiviral defense [165,166]
Key target pathogens	*A. hydrophila*, *V.anguillarum*/*harveyi*, *Flavobacterium* spp., *Streptococcus* spp.	*V. parahaemolyticus* (AHPND), *V. harveyi*, white spot syndrome virus (WSSV) [4,140]
Commonly applied probiotics	*Bacillus*, *Lactobacillus*, *Lactococcus*, *Enterococcus*, *Carnobacterium*, *Pediococcus*; yeast *Saccharomyces*	*Bacillus*, *Lactobacillus*, *Pseudoalteromonas*, *Leuconostoc*, *Streptococcus phocae* [68,96,140,159,160]
Typical efficacy readouts	Growth, IgT/IgM titers, cytokine expression, gut morphology, disease resistance	Growth, survival, phenoloxidase/proPO activity, hemocyte count, AMP expression, *Vibrio* load
Microbiome source and dynamics	Water and feed early; increasingly host-specific with age	Strong water/sediment contribution (up to ~85% early [31]); molt-associated shifts

## Data Availability

No new data were created or analyzed in this study. Data sharing is not applicable to this article.
